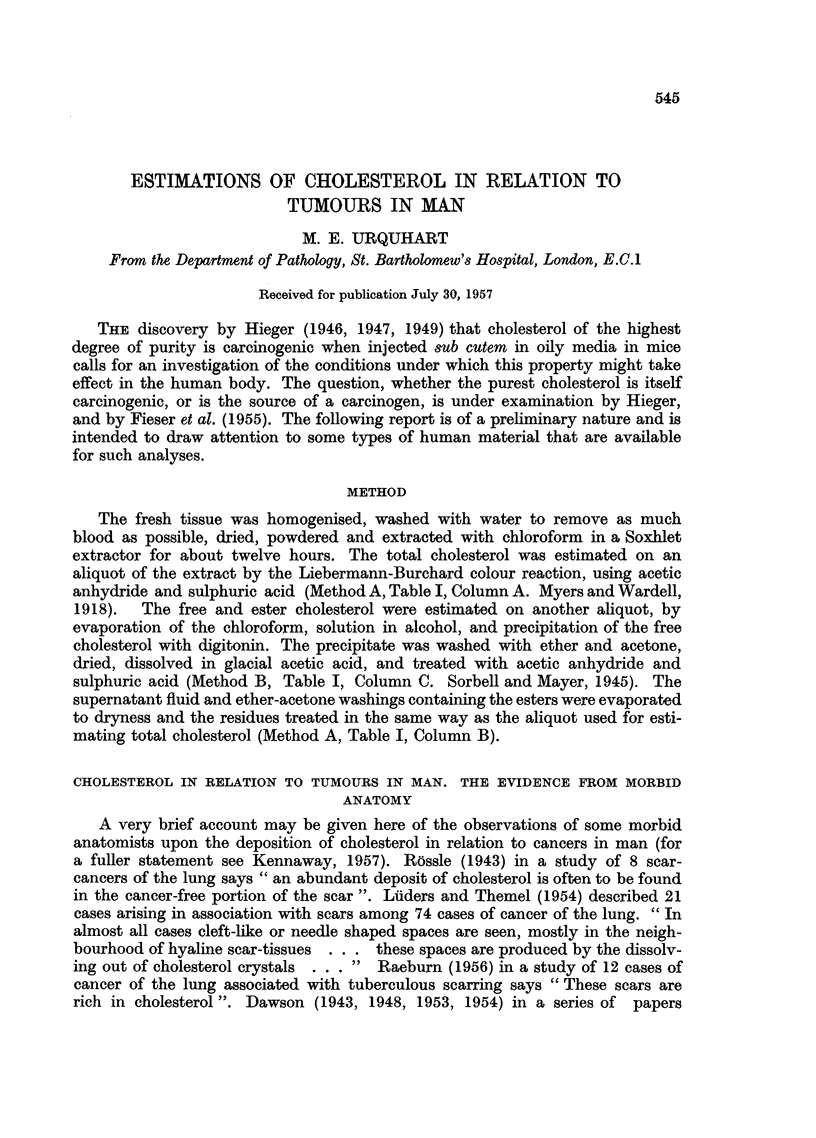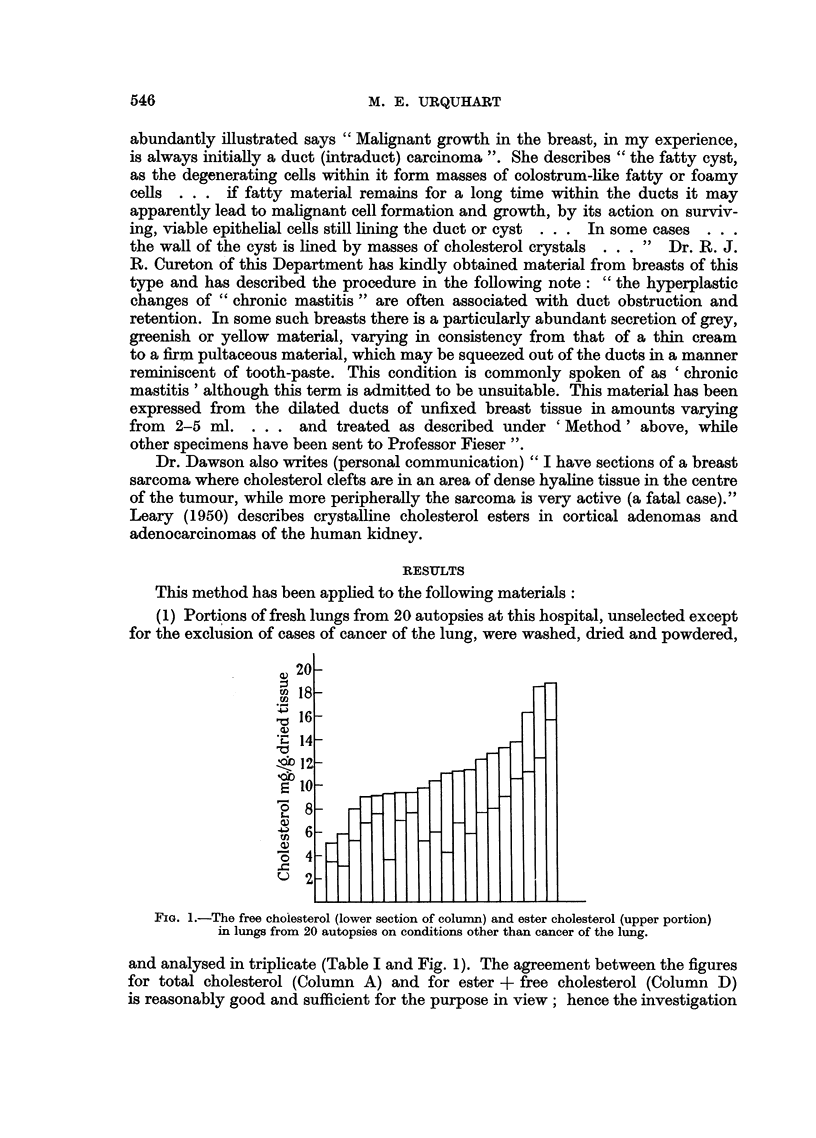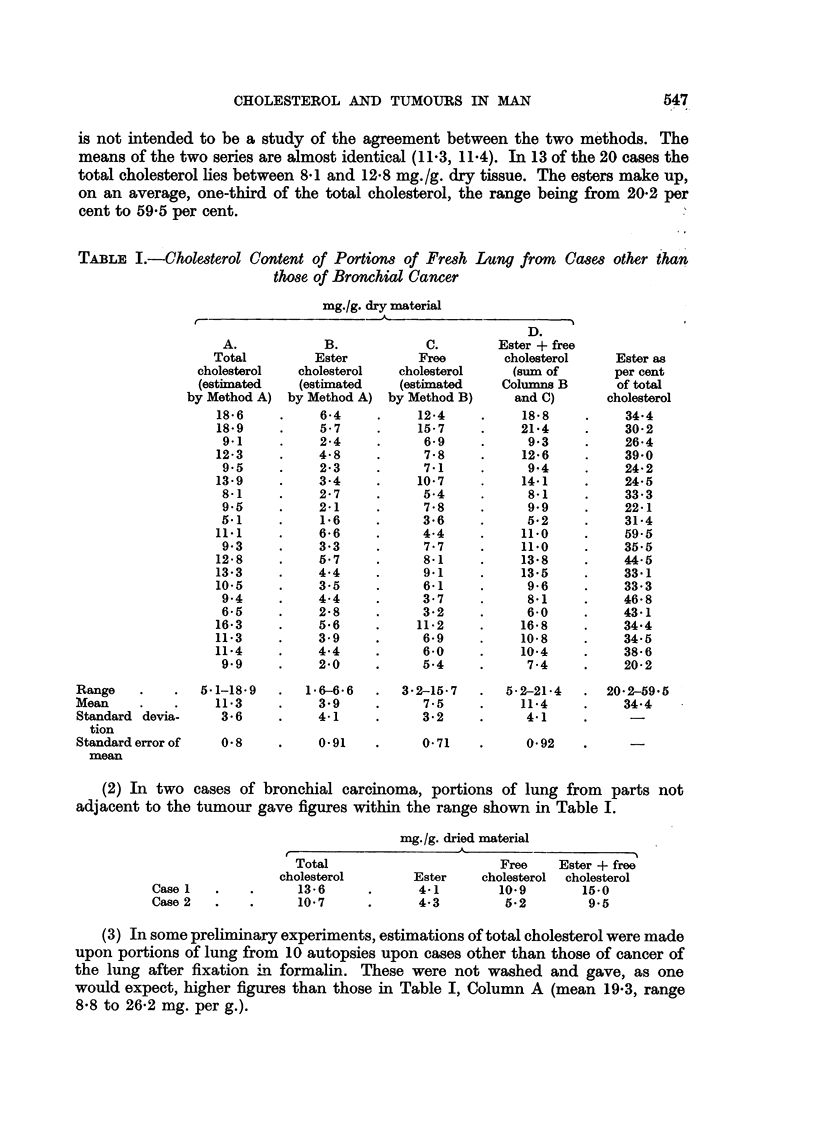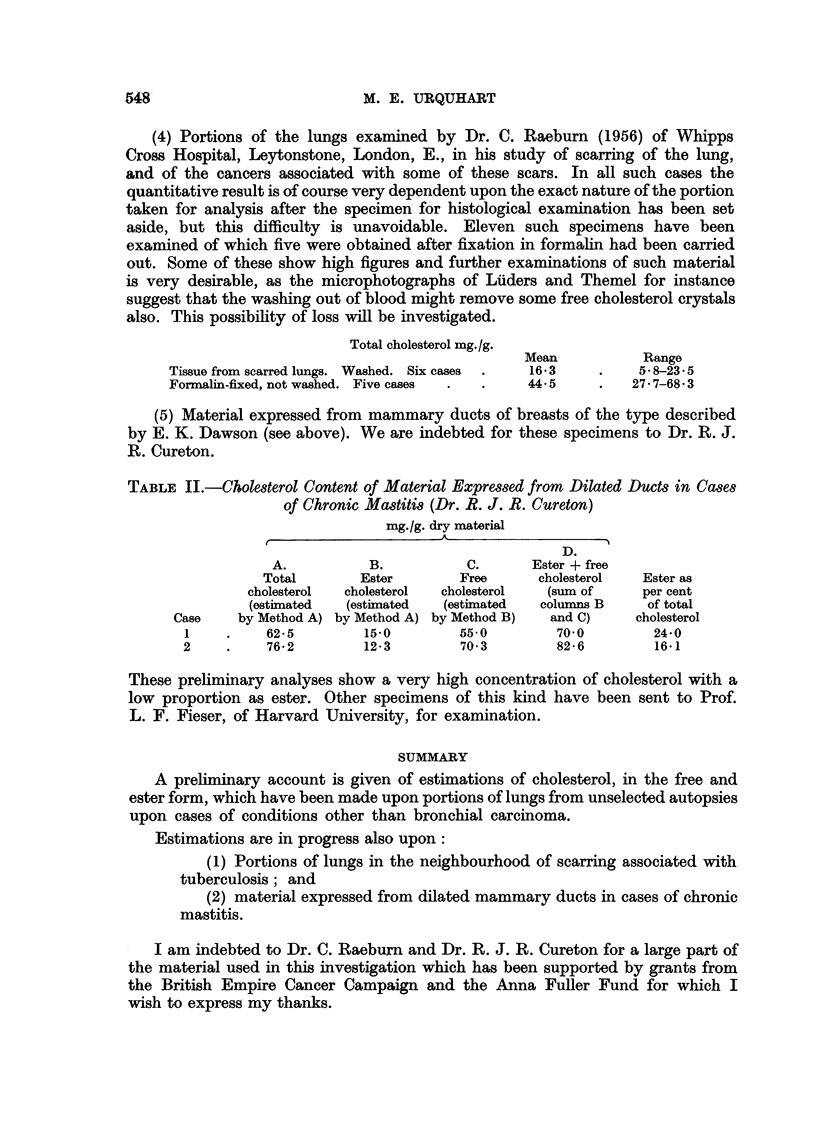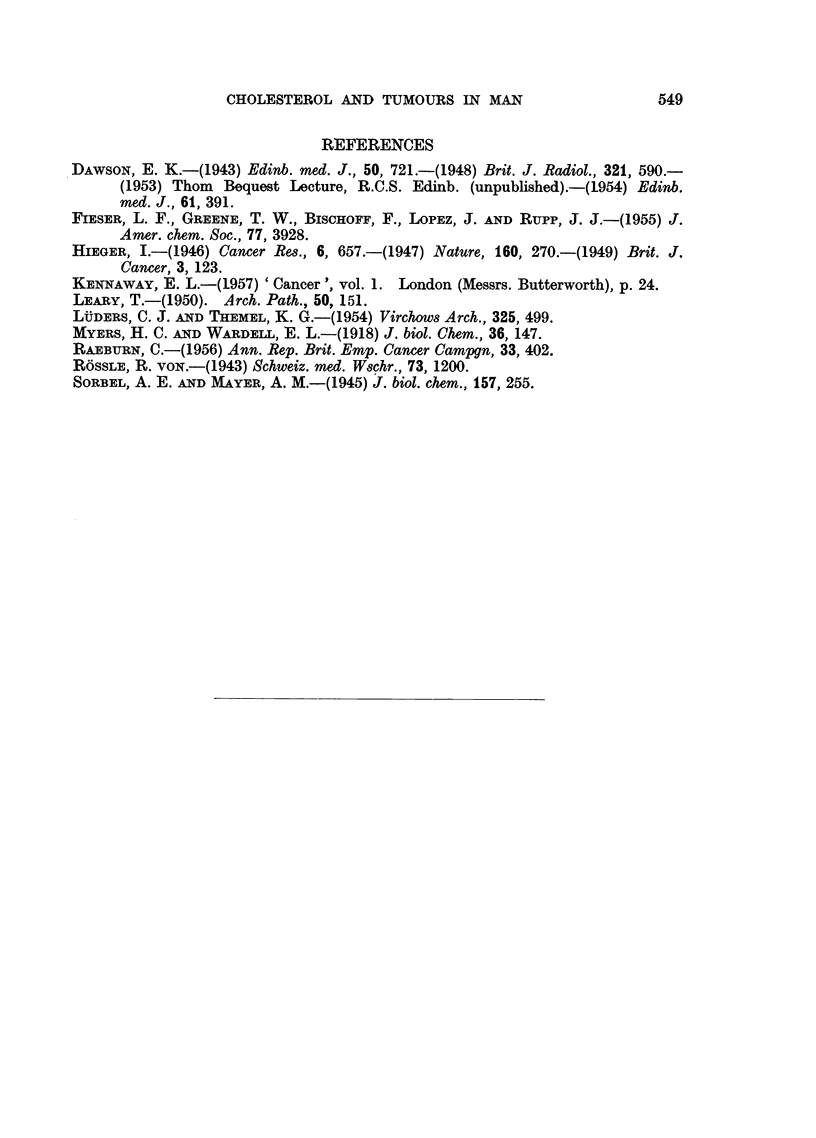# Estimations of Cholesterol in Relation to Tumours in Man

**DOI:** 10.1038/bjc.1957.66

**Published:** 1957-12

**Authors:** M. E. Urquhart


### ESTIMATIONS OF CHOLESTEROL IN RELATION TO TUMOURS IN MAN

M. E. URQUHART

*From the Department of Pathology, St. Bartholomew's Hospital, London, E.C.1*

Received for publication July 30, 1957

THE discovery by Hieger (1946, 1947, 1949) that cholesterol of the highest degree of purity is carcinogenic when injected *sub cutem* in oily media in mice calls for an investigation of the conditions under which this property might take effect in the human body. The question, whether the purest cholesterol is itself carcinogenic, or is the source of a carcinogen, is under examination by Hieger, and by Fieser *et al.* (1955). The following report is of a preliminary nature and is intended to draw attention to some types of human material that are available for such analyses.

### METHOD

The fresh tissue was homogenised, washed with water to remove as much blood as possible, dried, powdered and extracted with chloroform in a Soxhlet extractor for about twelve hours. The total cholesterol was estimated on an aliquot of the extract by the Liebermann-Burchard colour reaction, using acetic anhydride and sulphuric acid (Method A, Table I, Column A. Myers and Wardell, 1918). The free and ester cholesterol were estimated on another aliquot, by evaporation of the chloroform, solution in alcohol, and precipitation of the free cholesterol with digitonin. The precipitate was washed with ether and acetone, dried, dissolved in glacial acetic acid, and treated with acetic anhydride and sulphuric acid (Method B, Table I, Column C. Sorbell and Mayer, 1945). The supernatant fluid and ether-acetone washings containing the esters were evaporated to dryness and the residues treated in the same way as the aliquot used for estimating total cholesterol (Method A, Table I, Column B).

### CHOLESTEROL IN RELATION TO TUMOURS IN MAN. THE EVIDENCE FROM MORBID ANATOMY

A very brief account may be given here of the observations of some morbid anatomists upon the deposition of cholesterol in relation to cancers in man (for a fuller statement see Kennaway, 1957). Rössle (1943) in a study of 8 scar-cancers of the lung says " an abundant deposit of cholesterol is often to be found in the cancer-free portion of the scar ". Lüders and Themel (1954) described 21 cases arising in association with scars among 74 cases of cancer of the lung. " In almost all cases cleft-like or needle shaped spaces are seen, mostly in the neighbourhood of hyaline scar-tissues . . . these spaces are produced by the dissolving out of cholesterol crystals . . . " Raeburn (1956) in a study of 12 cases of cancer of the lung associated with tuberculous scarring says " These scars are rich in cholesterol ". Dawson (1943, 1948, 1953, 1954) in a series of papers abundantly illustrated says " Malignant growth in the breast, in my experience, is always initially a duct (intraduct) carcinoma ". She describes " the fatty cyst, as the degenerating cells within it form masses of colostrum-like fatty or foamy cells . . . if fatty material remains for a long time within the ducts it may apparently lead to malignant cell formation and growth, by its action on surviving, viable epithelial cells still lining the duct or cyst . . . In some cases . . . the wall of the cyst is lined by masses of cholesterol crystals . . . " Dr. R. J. R. Cureton of this Department has kindly obtained material from breasts of this type and has described the procedure in the following note : " the hyperplastic changes of " chronic mastitis " are often associated with duct obstruction and retention. In some such breasts there is a particularly abundant secretion of grey, greenish or yellow material, varying in consistency from that of a thin cream to a firm pultaceous material, which may be squeezed out of the ducts in a manner reminiscent of tooth-paste. This condition is commonly spoken of as ' chronic mastitis ' although this term is admitted to be unsuitable. This material has been expressed from the dilated ducts of unfixed breast tissue in amounts varying from 2–5 ml. . . . and treated as described under ' Method ' above, while other specimens have been sent to Professor Fieser ".

Dr. Dawson also writes (personal communication) " I have sections of a breast sarcoma where cholesterol clefts are in an area of dense hyaline tissue in the centre of the tumour, while more peripherally the sarcoma is very active (a fatal case)." Leary (1950) describes crystalline cholesterol esters in cortical adenomas and adenocarcinomas of the human kidney.

### RESULTS

This method has been applied to the following materials :

(1) Portions of fresh lungs from 20 autopsies at this hospital, unselected except for the exclusion of cases of cancer of the lung, were washed, dried and powdered,

FIG. 1.—The free cholesterol (lower section of column) and ester cholesterol (upper portion) in lungs from 20 autopsies on conditions other than cancer of the lung.

and analysed in triplicate (Table I and Fig. 1). The agreement between the figures for total cholesterol (Column A) and for ester + free cholesterol (Column D) is reasonably good and sufficient for the purpose in view ; hence the investigation is not intended to be a study of the agreement between the two methods. The means of the two series are almost identical (11·3, 11·4). In 13 of the 20 cases the total cholesterol lies between 8·1 and 12·8 mg./g. dry tissue. The esters make up, on an average, one-third of the total cholesterol, the range being from 20·2 per cent to 59·5 per cent.

TABLE I.—*Cholesterol Content of Portions of Fresh Lung from Cases other than those of Bronchial Cancer*

| | mg./g. dry material | | | | |
|---|---|---|---|---|---|
| | A. Total cholesterol (estimated by Method A) | B. Ester cholesterol (estimated by Method A) | C. Free cholesterol (estimated by Method B) | D. Ester + free cholesterol (sum of Columns B and C) | Ester as per cent of total cholesterol |
| | 18·6 | 6·4 | 12·4 | 18·8 | 34·4 |
| | 18·9 | 5·7 | 15·7 | 21·4 | 30·2 |
| | 9·1 | 2·4 | 6·9 | 9·3 | 26·4 |
| | 12·3 | 4·8 | 7·8 | 12·6 | 39·0 |
| | 9·5 | 2·3 | 7·1 | 9·4 | 24·2 |
| | 13·9 | 3·4 | 10·7 | 14·1 | 24·5 |
| | 8·1 | 2·7 | 5·4 | 8·1 | 33·3 |
| | 9·5 | 2·1 | 7·8 | 9·9 | 22·1 |
| | 5·1 | 1·6 | 3·6 | 5·2 | 31·4 |
| | 11·1 | 6·6 | 4·4 | 11·0 | 59·5 |
| | 9·3 | 3·3 | 7·7 | 11·0 | 35·5 |
| | 12·8 | 5·7 | 8·1 | 13·8 | 44·5 |
| | 13·3 | 4·4 | 9·1 | 13·5 | 33·1 |
| | 10·5 | 3·5 | 6·1 | 9·6 | 33·3 |
| | 9·4 | 4·4 | 3·7 | 8·1 | 46·8 |
| | 6·5 | 2·8 | 3·2 | 6·0 | 43·1 |
| | 16·3 | 5·6 | 11·2 | 16·8 | 34·4 |
| | 11·3 | 3·9 | 6·9 | 10·8 | 34·5 |
| | 11·4 | 4·4 | 6·0 | 10·4 | 38·6 |
| | 9·9 | 2·0 | 5·4 | 7·4 | 20·2 |
| Range | 5·1–18·9 | 1·6–6·6 | 3·2–15·7 | 5·2–21·4 | 20·2–59·5 |
| Mean | 11·3 | 3·9 | 7·5 | 11·4 | 34·4 |
| Standard deviation | 3·6 | 4·1 | 3·2 | 4·1 | — |
| Standard error of mean | 0·8 | 0·91 | 0·71 | 0·92 | — |

(2) In two cases of bronchial carcinoma, portions of lung from parts not adjacent to the tumour gave figures within the range shown in Table I.

| | mg./g. dried material | | | |
|---|---|---|---|---|
| | Total cholesterol | Ester | Free cholesterol | Ester + free cholesterol |
| Case 1 | 13·6 | 4·1 | 10·9 | 15·0 |
| Case 2 | 10·7 | 4·3 | 5·2 | 9·5 |

(3) In some preliminary experiments, estimations of total cholesterol were made upon portions of lung from 10 autopsies upon cases other than those of cancer of the lung after fixation in formalin. These were not washed and gave, as one would expect, higher figures than those in Table I, Column A (mean 19·3, range 8·8 to 26·2 mg. per g.).

(4) Portions of the lungs examined by Dr. C. Raeburn (1956) of Whipps Cross Hospital, Leytonstone, London, E., in his study of scarring of the lung, and of the cancers associated with some of these scars. In all such cases the quantitative result is of course very dependent upon the exact nature of the portion taken for analysis after the specimen for histological examination has been set aside, but this difficulty is unavoidable. Eleven such specimens have been examined of which five were obtained after fixation in formalin had been carried out. Some of these show high figures and further examinations of such material is very desirable, as the microphotographs of Lüders and Themel for instance suggest that the washing out of blood might remove some free cholesterol crystals also. This possibility of loss will be investigated.

| Total cholesterol mg./g. | Mean | Range |
| --- | --- | --- |
| Tissue from scarred lungs. Washed. Six cases | 16·3 | 5·8–23·5 |
| Formalin-fixed, not washed. Five cases | 44·5 | 27·7–68·3 |

(5) Material expressed from mammary ducts of breasts of the type described by E. K. Dawson (see above). We are indebted for these specimens to Dr. R. J. R. Cureton.

TABLE II.—*Cholesterol Content of Material Expressed from Dilated Ducts in Cases of Chronic Mastitis* (*Dr. R. J. R. Cureton*)

| | mg./g. dry material | | | | |
| --- | --- | --- | --- | --- | --- |
| Case | A. Total cholesterol (estimated by Method A) | B. Ester cholesterol (estimated by Method A) | C. Free cholesterol (estimated by Method B) | D. Ester + free cholesterol (sum of columns B and C) | Ester as per cent of total cholesterol |
| 1 | 62·5 | 15·0 | 55·0 | 70·0 | 24·0 |
| 2 | 76·2 | 12·3 | 70·3 | 82·6 | 16·1 |

These preliminary analyses show a very high concentration of cholesterol with a low proportion as ester. Other specimens of this kind have been sent to Prof. L. F. Fieser, of Harvard University, for examination.

### SUMMARY

A preliminary account is given of estimations of cholesterol, in the free and ester form, which have been made upon portions of lungs from unselected autopsies upon cases of conditions other than bronchial carcinoma.

Estimations are in progress also upon :

(1) Portions of lungs in the neighbourhood of scarring associated with tuberculosis ; and

(2) material expressed from dilated mammary ducts in cases of chronic mastitis.

I am indebted to Dr. C. Raeburn and Dr. R. J. R. Cureton for a large part of the material used in this investigation which has been supported by grants from the British Empire Cancer Campaign and the Anna Fuller Fund for which I wish to express my thanks.

### REFERENCES

DAWSON, E. K.—(1943) *Edinb. med. J.*, **50**, 721.—(1948) *Brit. J. Radiol.*, **321**, 590.—(1953) Thom Bequest Lecture, R.C.S. Edinb. (unpublished).—(1954) *Edinb. med. J.*, **61**, 391.

FIESER, L. F., GREENE, T. W., BISCHOFF, F., LOPEZ, J. AND RUPP, J. J.—(1955) *J. Amer. chem. Soc.*, **77**, 3928.

HIEGER, I.—(1946) *Cancer Res.*, **6**, 657.—(1947) *Nature*, **160**, 270.—(1949) *Brit. J. Cancer*, **3**, 123.

KENNAWAY, E. L.—(1957) ' Cancer ', vol. 1. London (Messrs. Butterworth), p. 24.

LEARY, T.—(1950). *Arch. Path.*, **50**, 151.

LÜDERS, C. J. AND THEMEL, K. G.—(1954) *Virchows Arch.*, **325**, 499.

MYERS, H. C. AND WARDELL, E. L.—(1918) *J. biol. Chem.*, **36**, 147.

RAEBURN, C.—(1956) *Ann. Rep. Brit. Emp. Cancer Campgn*, **33**, 402.

RÖSSLE, R. VON.—(1943) *Schweiz. med. Wschr.*, **73**, 1200.

SORBEL, A. E. AND MAYER, A. M.—(1945) *J. biol. chem.*, **157**, 255.